# Pathological Gaming and Its Association With Lifestyle, Irritability, and School and Family Environments Among Japanese Elementary School Children

**DOI:** 10.2188/jea.JE20210365

**Published:** 2023-07-05

**Authors:** Masaaki Yamada, Michikazu Sekine, Takashi Tatsuse

**Affiliations:** Department of Epidemiology and Health Policy, School of Medicine, University of Toyama, Toyama, Japan

**Keywords:** addiction, disorder, problematic, psychological, disease

## Abstract

**Background:**

Pathological gaming (PG) has emerged as one of the major public health concerns worldwide. We aimed to assess PG and its associated factors among elementary school children in Japan.

**Methods:**

We conducted a school-based observational study in Toyama, Japan in 2018. In total, 13,413 children in the 4th–6th grades (mean age, 10.5 years) participated in the study. We distributed questionnaires and inquired about their lifestyle, irritability, and school and family environments. Referring to criteria of gaming disorder in the International Statistical Classification of Disease (ICD-11), we asked about three core symptoms: impaired control over gaming, increasing priority given to gaming over other activities, and continuation of gaming despite the negative consequences. Children who had all three criteria in the questionnaire were defined as PG.

**Results:**

The response rate was 97.6%, and 11,826 children were included in our analyses (88.2%). The prevalence of PG was 5.6% (7.8% in boys, 3.2% in girls). Besides sex, PG was significantly associated with lifestyles, including skipping breakfast (odds ratio [OR] 1.33; 95% confidence interval [CI], 1.05–1.68), physical inactivity (OR 2.23; 95% CI, 1.63–3.05 for rare), late bed time (OR 2.52; 95% CI, 1.96–3.25 for ≥11 p.m.), frequent irritability (OR 1.89; 95% CI, 1.47–2.43), frequent feeling of school avoidance (OR 1.92; 95% CI, 1.49–2.46), fewer close friends (OR 1.30; 95% CI, 1.08–1.56 for some), low academic performance (OR 1.53; 95% CI, 1.13–2.08), no child–parent interaction (OR 1.34; 95% CI, 1.02–1.75), and no rules at home (OR 1.21; 95% CI, 1.02–1.43).

**Conclusion:**

Unhealthy lifestyles, irritability, and low functioning in school and family environments were associated with PG. Besides having a healthy lifestyle, parental involvement appears to be an indispensable countermeasure for PG in children.

## INTRODUCTION

In recent years, pathological gaming (PG) has become a public concern worldwide. Internet gaming disorder (IGD) was identified as a condition for further research in the Diagnostic and Statistical Manual of Mental Disorders, 5th Edition (DSM-5).^1^ In 2019, an expert group organized by the World Health Organization (WHO) confirmed gaming disorder as a mental disorder in the final version of International Classification of Diseases, 11th Version (ICD-11).^2^ In this classification, gaming disorder includes a pattern of persistent or recurrent online or offline gaming behavior manifested by the three core symptoms: (1) impaired control over gaming; (2) increasing priority given to gaming; and (3) continuation or escalation of gaming despite the occurrence of negative consequences in life.^3^ As gaming disorder is a new concept, more empirical studies are needed.

The prevalence rates of PG (or IGD, also referred to as game addiction) vary widely between studies from different countries, ranging from 0.7% to 27.5%.^4^ This may be caused by differences in assessment instruments, study populations, and diagnostic criteria for PG. Male sex, length of time spent playing games, sleep deprivation, family difficulties, low school performance, and personality issues, such as irritability and high impulsivity, have been reported as factors associated with PG.^4^^–^^7^

The number of studies on PG have been increasing; however, they are predominantly focused on junior high school-aged children or older adolescents. Although there have been several reports on PG among elementary school children, the scale of these studies was small to medium.^8^^,^^9^ Furthermore, a large-scale study targeting elementary school children is lacking. In Japan, the rate of internet use was approximately 85.6% among elementary school children, and 81.5% of the internet users reported that they played online games.^10^ Recent findings demonstrated that the initiation and habitual use of gaming at an early age is a strong risk factor for PG in later life.^11^^,^^12^ Elementary school age is a crucial period when young people develop their own lifestyle and may rapidly adopt gaming technology. Therefore, studies targeting elementary children are required for the early detection or educational intervention for PG.

We hypothesized that a large-scale epidemiological study assessing PG, which is a gaming disorder broadly defined based on the questionnaire, would be beneficial for researchers and medical staff, because they could understand the prevalence of possible gaming disorder before it becomes part of the ICD-11 in January 2022.^2^ Our aims were: (1) to report the prevalence of PG from a large-scale epidemiological study and (2) to elucidate its association with lifestyle, psychological status, and school and family environments among elementary school children in Japan.

## METHODS

### Participants: children from the Toyama Safe Internet Use Survey

Among elementary school children in Toyama Prefecture in Japan, a school-based cross-sectional study, the Toyama Safe Internet Use Survey, was conducted in 2018.^13^ Toyama Prefecture is located in the Chubu region of Honshu, Hokuriku Area, and has a population of approximately 1 million. From 185 in total, 110 elementary schools in Toyama Prefecture (as of 2018, 61.1% of elementary schools in Toyama) joined the survey, responding to a call by the Toyama Prefecture Education Board. In total, 13,413 children in the 4th–6th grades participated. We distributed an anonymous, self-reported questionnaire to all children in all schools. The purpose of this survey was explained by schoolteachers, and informed consent (assent) was acquired from children and their parents. Children answered and returned the questionnaire in the classroom. Participation was voluntary, and the parents (or guardians) were given the opportunity to opt out of the survey. The study was approved by the ethical review board of University of Toyama, and all methods were performed in accordance with the relevant guidelines and regulations.

### Measures

Our questionnaires contained information on basic characteristics, lifestyle, psychological status, school functioning and family environments, internet use, and PG. Lifestyle variables included consumption of breakfast, physical activity, and sleep habits. Responses to the breakfast question were dichotomized as “every day” or “skipping.” Physical activity responses included three levels: “very often,” “often,” or “rarely to almost never.” The validity of the lifestyle question asking about physical activity and sleep habits was examined in the previous studies and they demonstrated good agreement in subjective and objective measures.^14^^,^^15^ In those studies, frequent physical activity was significantly correlated with daily energy expenditure, mean steps, and mean activity count on the Actiwatch (*P* < 0.05 for a linear trend test). The correlation between subjective and objective records was 0.97 (*P* < 0.001) for assumed amount of sleep. To ascertain psychological status, questions covered frequency of feelings of irritability (“How often do you feel irritated?”) and self-esteem (“Do you like yourself?”).

In terms of the school functioning, information about the frequency of school avoidance (“How often do you feel like you are reluctant to go to school?”), close friends (“How many close friends do you have in real life?”), and subjective academic performance (“Do you understand school lectures well?”) was requested. The response of academic performance was on a five-point scale and collapsed into three: “high,” “middle,” and “low.” Subjective academic performance has previously been reported to be generally accurate,^16^ and has been used widely as a feasible surrogate variable.^17^^–^^19^ In terms of family environments, we asked about the setting of rules restricting screen time (for TV and DVD viewing, video-game playing, and recreational internet use) at home, and about child–parent interactions (“How often do you usually interact with your parents?”).

In assessing PG, we inquired about three core symptoms of gaming disorder (impaired control, priority to gaming, and continuation of gaming despite the negative consequences), following the ICD-11’s criteria on a 3-point Likert scale: “never”, “sometimes”, or “often.” Then, we divided the answers into two: no (never), and yes (sometimes or often). Children who answered “yes” for all three criteria were defined as having PG. The questionnaire on PG showed excellent reliability in this study (Cronbach’s α = 0.86). We also asked about internet time on weekdays. The responses were categorized into “<2 hours,” “2 to <3 hours,” “3 to <4 hours,” and “≥4 hours.”

We obtained children’s anthropometric data, which were measured by trained school nurses. Age- and sex-specific cutoff points equivalent to the adult body mass index (BMI) value of 25 or 18.5 kg/m^2^ for classification as overweight or underweight were used to identify children who were overweight or underweight, respectively. These cutoff points were developed by the Childhood Obesity Working Group of the International Obesity Task Force.^20^^,^^21^

### Statistical analysis

Descriptive analyses were performed on all variables and the prevalence of PG. After calculating the school-level intraclass correlation coefficients on PG (only 0.14%), a single-level logistic regression analysis was performed to clarify the factors associated with PG, and analyses were then stratified by sex. Crude and adjusted odds ratios (ORs and aORs, respectively) and 95% confidence intervals (CIs) were calculated. In the logistic regression analysis, we did not include internet time because PG and prolonged internet time seemed to be similar symptoms, and we previously reported the strong association between pathological internet use and internet time.^13^ In the multivariable analysis, all other independent variables were included simultaneously using the forced entry method. Finally, we examined the percentages of the three core symptoms of PG by internet time as a post-hoc test. Data analyses were performed using STATA version 14.0 (STATA Corporation, College Station, TX, USA) and SPSS version 25 (SPSS, Inc., Chicago, IL, USA). A two-tailed *P* value <0.05 was considered statistically significant.

## RESULTS

In total, 13,092 subjects returned the questionnaire (response rate 97.6%), and we included 11,826 (88.2%) children (mean age, 10.5; standard deviation, 0.96 years) who answered the relevant questionnaires in our analyses. The distributions of basic characteristics by sex are demonstrated in Table 1. More boys tended to get up late, be physically active, have higher self-esteem, have frequent feelings of school avoidance, and use the internet longer than girls do. Meanwhile, girls had more frequent interaction with their parents. The overall prevalence of PG was 5.6% (7.8% in boys, 3.2% in girls).

**Table 1.** Characteristics of children by sex, *n* = 11,826

		Boy	Girl	*P*
		% (*n* = 6,039)	% (*n* = 5,787)	Chi-square test
Grade	4	32.6	31.7	0.345
	5	32.8	34.0	
	6	34.5	34.4	
Wakeup time on weekdays	≥7:00	5.3	3.5	<0.001
Breakfast	Skipping	9.0	8.2	0.114
Physical activity	Very often	79.3	67.0	<0.001
	Often	17.3	28.5	
	Rarely	3.4	4.5	
Bedtime on weekdays	<10 p.m.	66.9	65.2	0.085
	10 to 11 p.m.	26.1	27.9	
	≥11 p.m.	7.0	6.9	
Irritability	Rare	35.3	32.7	0.009
	Sometimes	43.4	45.0	
	Often	21.3	22.3	
Self-esteem	High (I like myself)	66.6	61.8	<0.001
	Middle (not so much)	25.9	30.3	
	Low (not like)	7.4	7.9	
Feeling of school avoidance	Rare	57.2	62.0	<0.001
	Sometimes	30.0	29.7	
	Often	12.8	8.2	
Close friends	Many	64.8	64.6	0.309
	Some	29.0	29.8	
	Scarce or none	6.2	5.6	
Academic performance in school	High	86.9	86.9	0.523
	Middle	8.8	9.1	
	Low	4.4	4.0	
Interaction with parent	Often or sometime	68.5	84.7	<0.001
	Rare	21.5	12.0	
	None	10.0	3.2	
Setting rules for screen time	No	32.4	30.1	0.006
Body composition	Normal	73.2	72.6	<0.001
	Overweight	17.0	15.0	
	Underweight	9.7	12.4	
Internet time on weekdays (missing data: *n* = 43)	<2 h	61.0	74.9	<0.001
	2 h to <3 h	15.2	11.3	
	3 h to <4 h	9.7	5.4	
	≥4 h	14.1	8.3	
Pathological gaming		7.8	3.2	<0.001

Table 2 shows the results of a single-level logistic regression analysis, which was conducted to show the associations between children’s characteristics, lifestyle, psychological status, school functioning and family environments, body composition, and PG. In the multivariable analysis, boy (aOR 2.60; 95% CI, 2.15–3.13), late wakeup (≥7 a.m.: aOR 1.35; 95% CI, 1.01–1.81), skipping breakfast (aOR 1.33; 95% CI, 1.05–1.68), physical activity (often: aOR 1.38; 95% CI, 1.13–1.68 and rarely: aOR 2.23; 95% CI, 1.63–3.05), late bedtime (10 to 11 p.m.: aOR 1.60; 95% CI, 1.33–1.93 and ≥11 p.m.: aOR 2.52; 95% CI, 1.96–3.25), feeling of irritability (sometimes: aOR 1.40; 95% CI, 1.12–1.76 and often: aOR 1.89; 95% CI, 1.47–2.43), feelings of school avoidance (sometimes: aOR 1.75; 95% CI, 1.44–2.13 and often: aOR 1.92; 95% CI, 1.49–2.46), close friends in real life (some: aOR 1.30; 95% CI, 1.08–1.56), academic performance (middle: aOR 1.41; 95% CI, 1.11–1.78 and low: aOR 1.53; 95% CI, 1.13–2.08), interaction with parents (sometimes: aOR 1.34; 95% CI, 1.10–1.63 and rare or none: aOR 1.34; 95% CI, 1.02–1.75), and no rules at home (aOR 1.21; 95% CI, 1.02–1.43) were significantly associated with PG (Table 2).

**Table 2.** Logistic regression analyses on pathological gaming, *n* = 11,826

		Pathological gaming	Univariable		Multivariable	
		%	OR (95% CI)	*P*	aOR (95% CI)	*P*
Sex boys/girls		7.8/3.2	**2.51 (2.11**–**2.99)**	**<0.001**	**2.60 (2.15–3.13)**	**<0.001**
Grade	4	4.9	1		1	
	5	4.8	0.98 (0.80–1.21)	0.869	0.88 (0.71–1.09)	0.233
	6	6.9	**1.43 (1.18–1.73)**	**<0.001**	1.16 (0.94–1.42)	0.164
Wakeup time on weekdays	≥7:00/<7:00	13.1/5.2	**2.74 (2.10–3.58)**	**<0.001**	**1.35 (1.01–1.81)**	**0.046**
Breakfast	Skipping/every day	11.7/5.0	**2.54 (2.06–3.13)**	**<0.001**	**1.33 (1.05–1.68)**	**0.017**
Physical activity	Very often	4.7	1		1	
	Often	6.9	**1.50 (1.25–1.79)**	**<0.001**	**1.38 (1.13–1.68)**	**0.001**
	Rarely	14.3	**3.40 (2.58–4.48)**	**<0.001**	**2.23 (1.63–3.05)**	**<0.001**
Bedtime on weekdays	<10 p.m.	3.8	1		1	
	10 to 11 p.m.	7.2	**1.95 (1.63–2.33)**	**<0.001**	**1.60 (1.33–1.93)**	**<0.001**
	≥11 p.m.	15.7	**4.68 (3.75–5.83)**	**<0.001**	**2.52 (1.96–3.25)**	**<0.001**
Irritability	Rare	3.1	1		1	
	Sometimes	5.3	**1.76 (1.42–2.19)**	**<0.001**	**1.40 (1.12–1.76)**	**0.004**
	Often	10.1	**3.56 (2.85–4.43)**	**<0.001**	**1.89 (1.47–2.43)**	**<0.001**
Self-esteem	High	4.6	1		1	
	Middle	6.5	**1.44 (1.21–1.72)**	**<0.001**	0.98 (0.81–1.18)	0.831
	Low	10.2	**2.34 (1.84–2.98)**	**<0.001**	0.96 (0.73–1.27)	0.783
Feeling of school avoidance	Rare	3.2	1		1	
	Sometimes	7.8	**2.55 (2.13–3.06)**	**<0.001**	**1.75 (1.44–2.13)**	**<0.001**
	Often	12.7	**4.43 (3.58–5.48)**	**<0.001**	**1.92 (1.49–2.46)**	**<0.001**
Close friends	Many	4.3	1		1	
	Some	7.5	**1.79 (1.52–2.12)**	**<0.001**	**1.30 (1.08–1.56)**	**0.005**
	Scarce or none	9.7	**2.38 (1.81–3.13)**	**<0.001**	1.19 (0.88–1.62)	0.261
Academic performance in school	High	4.8	1		1	
	Middle	11.8	**2.43 (1.99–2.99)**	**<0.001**	**1.41 (1.11–1.78)**	**<0.001**
	Low	13.6	**3.10 (2.39–4.02)**	**<0.001**	**1.53 (1.13–2.08)**	**<0.001**
Interaction with parent	Often or sometime	4.3	1		1	
	Rare	8.7	**2.15 (1.78–2.59)**	**<0.001**	**1.34 (1.10–1.63)**	**0.004**
	None	12.5	**3.23 (2.55–4.08)**	**<0.001**	**1.34 (1.02–1.75)**	**0.033**
Setting rules for screen time	No/yes	8.1/4.4	**1.91 (1.63–2.24)**	**<0.001**	**1.21 (1.02–1.43)**	**0.033**
Body composition	Normal	5.4	1		1	
	Overweight	7.4	**1.42 (1.17–1.73)**	**<0.001**	1.09 (0.88–1.34)	0.441
	Underweight	4.2	0.78 (0.58–1.04)	0.084	0.89 (0.66–1.20)	0.437

aOR, adjusted odds ratio; CI, confidence interval; OR, odds ratio.

Table 3 shows the difference between boys and girls from the multivariable logistic regression analyses on PG. Although trends were similar in general, unhealthy lifestyle practices, such as late wakeup, skipping breakfast, late bedtime, and physical inactivity, and infrequent interaction with parents had higher aORs in girls, while lower academic performance had higher aOR in boys (Table 3).

**Table 3.** Logistic regression analyses on pathological gaming by sex, *n* = 11,826

		Boy			Girls		

		Pathological gaming	Multivariable		Pathological gaming	Multivariable	
		%	aOR (95% CI)	*P*	%	aOR (95% CI)	*P*
Grade	4	7.0	1		2.7	1	
	5	7.1	0.92 (0.72–1.19)	0.522	2.6	0.74 (0.49–1.11)	0.147
	6	9.3	1.16 (0.91–1.47)	0.242	4.4	1.08 (0.73–1.59)	0.711
Wakeup time on weekdays	≥7:00/<7:00	14.2/7.4	1.13 (0.79–1.62)	0.491	11.4/3.0	**2.26 (1.35**–**3.79)**	**0.002**
Breakfast	Skipping/every day	13.6/7.2	1.21 (0.91–1.62)	0.196	9.7/2.7	**1.53 (1.02**–**2.28)**	**0.040**
Physical activity	Very often	6.7	1		2.2	1	
	Often	10.8	**1.33 (1.04–1.69)**	**0.023**	4.4	**1.49 (1.06–2.10)**	**0.021**
	Rarely	18.4	**1.91 (1.27–2.88)**	**0.002**	11.1	**2.88 (1.76–4.72)**	**<0.001**
Bedtime on weekdays	<10 p.m.	5.7	1		1.9	1	
	10 to 11 p.m.	10.5	**1.56 (1.25–1.95)**	**<0.001**	4.0	**1.82 (1.27–2.61)**	**0.001**
	≥11 p.m.	17.7	**2.00 (1.46–2.75)**	**<0.001**	13.6	**4.01 (2.60–6.18)**	**<0.001**
Irritability	Rare	4.4	1		1.6	1	
	Sometimes	8.0	**1.53 (1.18–1.99)**	**0.001**	2.5	1.10 (0.70–1.75)	0.671
	Often	13.0	**1.77 (1.32–2.37)**	**<0.001**	7.2	**2.04 (1.26–3.30)**	**0.004**
Self-esteem	High	6.7	1		2.2	1	
	Middle	8.9	0.91 (0.73–1.14)	0.412	4.3	1.12 (0.79–1.60)	0.512
	Low	13.1	0.95 (0.68–1.34)	0.772	7.2	0.92 (0.55–1.54)	0.755
Feeling of school avoidance	Rare	4.7	1		1.8	1	
	Sometimes	10.4	**1.76 (1.40–2.22)**	**<0.001**	4.9	**1.72 (1.19–2.48)**	**0.004**
	Often	15.4	**1.97 (1.48–2.63)**	**<0.001**	8.4	**1.75 (1.06–2.89)**	**0.029**
Close friends	Many	6.1	1		2.5	1	
	Some	10.6	**1.42 (1.14–1.75)**	**0.001**	4.3	1.09 (0.77–1.54)	0.619
	Scarce or none	12.5	1.21 (0.83–1.75)	0.322	6.4	1.28 (0.73–2.22)	0.388
Academic performance in school	High	6.5	1		2.8	1	
	Middle	14.9	**1.60 (1.21–2.12)**	**0.001**	5.9	0.97 (0.62–1.51)	0.875
	Low	18.6	**1.84 (1.28–2.65)**	**0.001**	7.8	0.99 (0.55–1.78)	0.969
Interaction with parent	Often or sometime	6.4	1		2.5	1	
	Rare	10.0	1.20 (0.95–1.52)	0.119	6.3	**1.80 (1.22–2.64)**	**0.003**
	None	12.8	1.20 (0.89–1.63)	0.228	11.7	**2.36 (1.35–4.15)**	**0.003**
Setting rules for screen time	No/yes	11.2/6.2	**1.32 (1.08–1.62)**	**0.007**	4.7/2.6	0.98 (0.71–1.36)	0.909
Body composition	Normal	7.4	1		3.2	1	
	Overweight	9.9	1.13 (0.89–1.45)	0.320	4.5	0.96 (0.65–1.42)	0.841
	Underweight	6.8	0.95 (0.67–1.35)	0.790	2.1	0.79 (0.45–1.39)	0.412

OR, odds ratio; aOR, adjusted odds ratio; CI, confidence interval.

**Figure 1.**
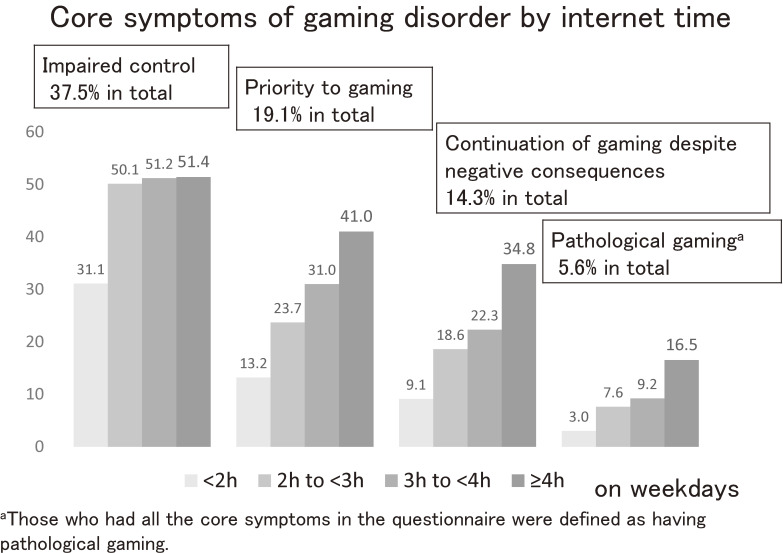
Frequency of the three core symptoms of gaming disorder by internet time on weekdays. In total, 37.5% of children report feelings of impaired control, 19.1% report giving increased priority to gaming over other activities, and 14.3% report the continuation of gaming despite the negative consequences in real life. Half of the children using the internet for ≥2 h per day report difficulty in controlling the time. In terms of other symptoms of gaming disorder, these gradually increase as the internet time increases.

Figure 1 demonstrates the percentage of each of the three core symptoms of gaming disorder in the ICD-11 by internet time on weekdays. In total, 37.5% of children experienced a feeling of impaired control, 19.1% of them reported increasing priority given to gaming over other activities, and 14.3% of them reported continuation of gaming despite the negative consequences in real life. Half of children using the internet for ≥2 hours reported difficulty in controlling the time. In terms of other symptoms and PG, gradual increases were seen as the internet time increased (Figure 1).

## DISCUSSION

There have hardly been any large-scale studies assessing PG among elementary school children. Our large-scale epidemiological study has helped gain valuable information on PG, a gaming disorder broadly defined using a questionnaire. In total, the prevalence of PG was 5.6%, and it was significantly associated with boys; unhealthy lifestyles, such as late wakeup and bedtime, skipping breakfast, and physical inactivity; frequent irritability; frequent feelings of school avoidance; fewer close friends; lower academic performance; insufficient child–parental interaction; and no rules at home.

In this study, we found a prevalence of PG of 5.6% among Japanese elementary school children, which is equivalent to more than one in 20 children. The studies on the prevalence of PG (or IGD) have previously utilized a wide range of different questionnaires, such as the DSM-5, Game Addiction Scale (GAS),^22^ and Young’s diagnostic questionnaire (YDQ).^23^ Paulus et al^6^ reported that prevalence varies widely, ranging from 0.6% in Norway to 50% in Korea. Fam’s meta-analysis reported a pooled prevalence of 4.6% overall and 9.9% in Asia.^24^ Comparing our findings with other studies seems difficult because of the differing age of participants and criteria; however, this percentage is a warning for parents, teachers, and health providers, especially in Asian countries.

We showed that PG was significantly associated with unhealthy lifestyles, including late wakeup and bedtime on weekdays, skipping breakfast, and physical inactivity, which is consistent with previous reports.^6^^,^^25^^,^^26^ Prolonged gaming may be taking time away from sleep and exercise. According to the concept of “life course epidemiology,” lifestyles during childhood have a long-term effect on later health or disease risk.^27^ To develop a regular healthy lifestyle in children, strict routines, such as going to bed before 10 p.m. and regular exercise habits, should be encouraged.

Many studies have shown the association between IGD and psychological health aspects, including impulsivity, irritability, physical aggression, and low self-esteem.^4^^,^^5^^,^^28^ Similarly, frequent feelings of irritability were associated with PG in our study. However, self-esteem was not associated with PG in the multivariable model, though the association was significant in the univariable analysis. The two plausible reasons for the non-significance in our study were as follows: 1) there might be other confounding factors in our analysis, such as feelings of school avoidance and number of close friends; and 2) self-esteem in elementary school children with PG might not be lower than that in adolescents. In any case, parents should learn about the risks of psychological health problem before allowing children to partake in gaming.

Consistent with our findings, other studies have also shown that social factors (or school factors in children) are associated with IGD.^4^^,^^5^^,^^29^ Frequent feelings of school avoidance, fewer close friends in real life, and low academic performance were significantly associated with PG. As we wanted to assess the importance of “off-line friends,” our survey specified close friends only in real life. Although one study found that IGD was associated with having more close friends,^30^ participants were not asked whether the close friends were online or real-life friends. Online friends tend to seek other friends with similar interests (gaming), while having real-life friends is associated with a decreased risk of PG. Distinguishing between online or off-line friends is important in epidemiological studies. Regarding intervention strategies, developing social skills from an early age is important for PG prevention. Several randomized controlled trials have demonstrated that use of a play-based intervention or other psychotherapies, including Program for the Education and Enrichment of Relational Skills (PEERS) or Children’s Friendship Training (CFT), have improved children’s social skills, such as communication and coping skills.^31^^–^^34^ We inferred that developing social skills, particularly while in elementary school or at an even younger age, would be important and that it could enable children to develop “off-line friendships” throughout their lives. Regarding academic performance, we found that lower subjective academic performance was associated with PG, which is consistent with other studies.^4^^,^^29^ These associations have been explored mainly in junior high or older school children. Our study demonstrated that this association can also apply to elementary school children. Our study cannot mention the direction of causality between PG and low academic performance, but establishing study habits or attending cram schools may ameliorate risk of PG.

We revealed that family environments, including child–parent interaction and rules for restricting screen time at home, had a significant association with PG. Our findings accorded with that of previous studies stating that family difficulties and disharmony were associated with an increased prevalence of IGD.^4^^,^^6^^,^^35^ Regarding rules at home, there have been several studies showing the association between a child’s screen time and rules at home.^36^^,^^37^ However, results of a study on the association between rules and IGD were inconclusive.^12^ Schneider et al^38^ insisted that parental restriction of screen time was only effective when there was joint agreement on the rules. We previously reported the importance of restricting parental internet use to less than 2 hours to prevent children’s prolonged screen time.^39^ Sufficient child–parent interaction and parental engagement may be essential strategies for PG prevention in children.

In our study, boys were more likely to have PG than girls (7.8% vs 3.2%). This is in line with other studies.^6^ Males are known to play more frequently and play more violent games than do females, often for excitement and for making friends online. Meanwhile, females engage in gaming to pass time, for social networking, and for texting. In our stratified analysis by sex (Table 3), boys had higher aOR in lower academic performance, while girls had fewer interaction with parents than boys. In addition to promoting healthy lifestyles in general, sex-specific measures should be taken.

As an ad hoc test, we demonstrated the percentage of children having core symptoms of gaming disorder in the ICD-11 by internet time (Figure 1). Gradual increases in symptoms are seen in terms of continuation of gaming despite the negative consequences in real life as internet time increases, while half of children who use the internet for ≥2 hours had a sense of impaired control. This manifests as difficulties in self-control in internet use in children. Parents seriously need to know the strong appeal that internet contents have for children.

The strengths of our study include its large-scale elementary school population and the high response rate (97.6%); however, our study also had several limitations. First, we assessed PG following the criteria for gaming disorder in the ICD-11; however, our questionnaire did not include the duration of gaming disorder because of children’s fluctuating behaviors. Moreover, gaming disorder in the ICD-11 was designed to be used in clinical settings. Therefore, our questionnaire-based survey on PG might show a higher prevalence than that of gaming disorder diagnosed through medical interviews. Discordance between the self-report and clinical diagnosis of IGD has been reported among Korean adolescents. Jeong et al^40^ found that 44% of the responses were false-negative (ie, negative in self-report and positive in clinical diagnosis). Therefore, the prevalence of PG in our study might not differ significantly from that of gaming disorder diagnosed based on the ICD-11. Nevertheless, the findings from our large-scale study are useful for school and medical staff. Second, because our study was a cross-sectional design, causality could not be elucidated. However, paying attention to children’s lifestyles and increasing child–parent interactions can be useful in any case for prevention or early detection of PG. Third, information on other mental disorders, such as attention deficit hyperactivity disorder and autism spectrum disorder, was not included. Finally, in our study, all participants were from one prefecture, Toyama, in Japan. Thus, whether our findings can be generalized is unclear. Future studies should address these concerns.

In conclusion, our large-scale survey revealed that the prevalence of PG was 5.6% in elementary school children, and it was associated with boys, unhealthy lifestyles, frequent irritability, and low functioning in school and family environments. Besides having a healthy lifestyle, parental involvement appears to be an indispensable countermeasure for PG in children.
